# Parents' pandemic stress, parental involvement, and family quality of life for children with autism

**DOI:** 10.3389/fpubh.2022.1061796

**Published:** 2022-12-01

**Authors:** Shengli Cheng, Sanyin Cheng, Shushan Liu, Yun Li

**Affiliations:** School of Philosophy and Social Development, Shandong University, Jinan, China

**Keywords:** family quality of life (FQOL), pandemic stress, parental involvement, children with autism, China

## Abstract

**Background:**

Research has shown that parents of children with autism spectrum disorder (ASD) suffered high levels of stress during the COVID-19 pandemic and faced poor family quality of life (FQOL). However, little is known about the inherent dynamic interaction between pandemic stress and FQOL, especially in the Chinese cultural context.

**Aims:**

This study provides preliminary evidence by examining the relationships among pandemic stress, parental involvement, and FQOL for children with autism in mainland China.

**Method:**

A total of 709 parents of children with autism completed measures of FQOL, parental involvement, and pandemic stress. Structural equation modeling was employed to examine the interrelations among these variables.

**Results:**

Pandemic stress has direct effect and indirect effect mediated by parental involvement on FQOL. Two dimensions of pandemic stress had a direct effect on FQOL (β1 = 0.11; β2 = −0.55) and three dimensions had an indirect effect on FQOL through parental involvement (β1 = −0.097; β2 = 0.257; β3 = 0.114).

**Conclusion:**

Stress related to the COVID-19 pandemic affects family quality of life for children with autism in complex ways. Policies may be developed to enhance parental *pragmatic hopefulness* in the anti-epidemic victory and alleviate negative *physical and mental reactions* caused by the pandemic.

## Introduction

It has been almost 3 years since the novel coronavirus was first discovered and the COVID-19 pandemic began in Wuhan, China, and so far, the pandemic shows no signs of ending. The pandemic has resulted in numerous adjustments to daily life for children and their caregivers, including children with ASD (autism spectrum disorder) and their parents. ASD is a pervasive developmental disability characterized by social-communication and interaction deficits, restricted and repetitive patterns of behavior, and significant functional impairments (1). Providing care for children with autism exposes their families to high levels of psychological stress and a lower quality of life for families (2). The adjustments to the COVID-19 pandemic, such as stay-at-home orders and remote learning, have impacted caregivers' wellbeing (3, 4) and, in the case of families of children with autism, further reduced family quality of life (FQOL) (5). Although studies have shown that FQOL for children with autism was affected by the COVID-19 pandemic (5), few have examined COVID-19's impact on families with autistic children from a multifactorial holistic perspective. This study provides preliminary evidence of the relationships among pandemic stress, parental involvement, and FQOL for children with autism in mainland China.

### Studies concerning family quality of life for children with autism

The concept of FQOL has been used to assess family adjustment outcomes for children with autism and is increasingly attracting the attention of researchers worldwide (6, 7). Several studies have shown that FQOL for children with autism is lower than for families of children with other disabilities (8, 9), making the study of FQOL for children with autism particularly important for social welfare.

Previous research has focused on exploring children with autism's overall FQOL and its possible predictors. These predictors focus on the child with ASD's: (1) individual level (6, 7, 10), (2) family level (6, 7, 11–14), and (3) social support level (15, 16).

The severity of symptoms in children with autism negatively predicts their FQOL (6, 7, 10). In addition to social support (15, 16), family cohesion (7), parental stress (11), and parental involvement (12) are associated with FQOL, indicating it is the result of multiple factors.

Although there is still a lack of representative large-scale epidemiological surveys on children with autism in China, the number of children being diagnosed with autism is increasing. Meanwhile, Chinese families experience high stress levels and low FQOL (17). These families experience high levels of parenting stress, financial burden, and limited family support (18). Hence, further investigating FQOL among children with autism in China is worthwhile.

### Parental stress related to ASD and its association with FQOL

Previous findings suggested that parental stress is greater for parents of children with autism than for those of typically developing children and children with other disability types (19–21). In the existing studies, parental stress, as an independent variable, affects the lives and growth of many children with autism and their parents. The relationship between parental stress and FQOL has received much attention, and some studies have demonstrated parental stress' lasting impact on children with autism's FQOL (22).

The current research on parental stress related to ASD and FQOL comprises three main aspects. The first aspect concerns their current state; parents of children with autism tend to have higher levels of parental stress and lower levels of FQOL [e.g., (8, 23, 24)]. The second aspect concerns the outcome assessments of parental stress, with some studies involving FQOL as an important parental stress outcome in assessing autistic children's families' overall satisfaction [e.g., (9)].

The third aspect regards their causal analysis. For instance, Likhitweerawong et al. (25) identified that, among 61 and 63 Thai caregivers of children with and without ASD, respectively, higher parental stress correlated moderately with lower authoritative, higher authoritarian, and higher permissive parenting styles, while a negative correlation was found between authoritarian and permissive parenting styles and children with ASD's quality of life. Pozo et al. (7) found that among 118 Spanish parents (59 mothers and 59 fathers) with a child with ASD, behavior problems negatively affected FQOL indirectly (through sense of coherence). The severity of the disorder and social support levels played significant roles in FQOL models for both fathers and mothers, whereas coping played differentiated roles in their FQOL. Through a meta-analysis of 29 studies (*N* = 4,864), Wang et al. (26) found that among caregivers for autism, social support partially mediated the relationship between coping (positive and negative) and family quality of life.

During the COVID-19 pandemic, people have stayed home and socially isolated themselves to avoid contracting the virus (27). Existing studies have demonstrated that while isolation somewhat reduces the risk of virus transmission, it also brings anxiety and psychological stress (28–30). In particular, parents affected by the pandemic who are raising infants or children with disabilities receive lower levels of social support and are at greater risk of psychological distress (3, 31–33).

The relationship between parental stress and FQOL is of great academic interest. Studies exploring the relationship between the two could help to reduce stress and improve FQOL for children with autism and their families during COVID-19. Some studies have analyzed parental stress as an influential factor in family relationships, regulation, and social support in the COVID-19 epidemic context (34–36). However, to the authors' best knowledge, no studies have focused on the relationship between pandemic stress and FQOL of children with autism. Thus, identifying and discussing the role of COVID-19-pandemic-related stress in parents of children with autism's FQOL is worthwhile.

### Parental involvement as an intermediary

Research on parental involvement originated with Englund et al.s' (37) study, which noted a positive relationship between parental involvement and children's school performance. Existing studies summarize the basic content and common forms of parental involvement, which mainly include the child's education plan, intervention plan, and educational career planning (38–40), as well as participation after communication with the intervention team and participation with the child in implementing the program (41, 42).

Parents need to spend more time and energy caring for children with autism, so it is essential to study parental involvement and related factors, which are important parts of developmental and therapeutic strategies for children with autism and particularly critical in the child's early development, education, and therapeutic interventions (43, 44). Parental involvement with children with autism covers a wide range of areas, including participating in the child's development, learning, and treatment and actively interacting with teachers and physicians (45, 46). Increasingly, parents of children with autism are becoming involved in their child's activities and interventions, participating in homework tutoring, parent training, and the design and implementation of intervention processes (29, 47–49). In this process, it is possible to gain a more comprehensive understanding of the effectiveness of engagement behaviors and thus better design and implement them when considering family outcomes, using FQOL as an indicator (50, 51).

It has been confirmed that parental involvement in the care and education of children with autism can have a positive impact on the child's behavioral styles, character personality, future development, and family relationships and interactions (52–54). Some studies have focused on the impact of parental psychological stress on parental involvement in children with autism, showing that increased parenting stress and decreased supportive behaviors and child care lead to decreased parental involvement (55, 56).

Some existing studies have confirmed the relationship between parental stress and involvement (57, 58), while others have demonstrated that parental involvement can influence FQOL (12, 59). In China, studies have found that parents of children with autism have higher parental psychological stress and are less actively involved in parenting than parents of normal children and children with other disabilities (60, 61). Thus, it is valuable to explore whether parental involvement mediates the relationship between pandemic stress and FQOL.

### The present research

The purpose of this study was to enrich the research on the relationships among pandemic stress, parental involvement, and FQOL for children with autism. Two research questions were proposed: (1) What are the current status of pandemic stress, parental involvement, and FQOL for children with autism in China? (2) What are the relationships among pandemic stress, parental involvement, and FQOL? Based on previous studies, two hypotheses were made. The first was that pandemic stress would directly predict FQOL. The second was that pandemic stress would indirectly predict FQOL, mediated by parental involvement.

**Figure 1:**
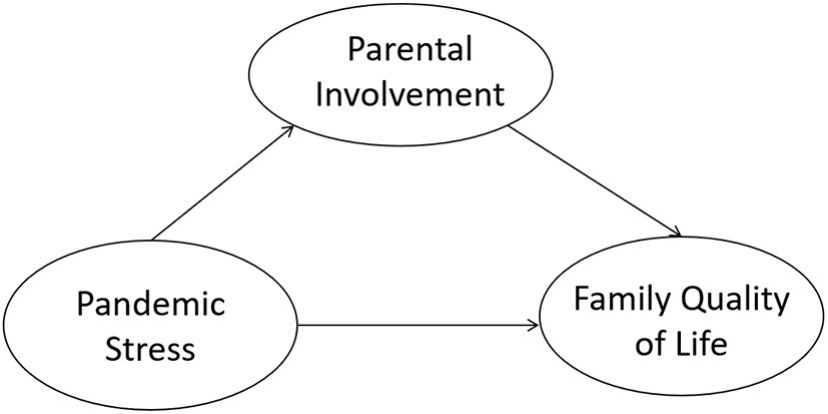
Hypothetical model.

A review of the current literature and research hypotheses suggested that pandemic stress would predict FQOL through direct and indirect pathways, with parental involvement mediating the latter. This study's proposed hypothetical model is shown in Figure 1.

## Methods

### Procedures

As the study did not have direct access to a list of children with autism in China, it used schools as a hub to introduce the online questionnaire to principals of special public schools serving children under the age of 22 with autism, in 31 provinces, autonomous regions, and municipalities. The principals then introduced and distributed the questionnaire to their students' parents, encouraging them to participate in this research.

All participants in the study completed the questionnaire online, which usually took about 20 min. All participants uploaded an informed consent form stating that participation was voluntary and that all information collected through the questionnaire would be kept strictly confidential and used for academic research only. All demographic information was anonymized. Upon completing the questionnaire, participants were randomly given an online bonus package, with a one-third chance of winning, to show the researchers' appreciation for their participation. It is worth noting that the data was collected when children were able to attend classes a bit in person/hybrid learning.

### Participants

Eight hundred and one families of children with autism completed the survey. Since the present research target children with autism, and those with autism usually lag behind the normal population across diverse developmental domains, we selected families with children aged ≤22 years as our sample, resulting in a final sample of 761 families. Removing outliers left 709 participants, 16% male and 84% female. Parents were mostly 31–50 years old. Their education was mostly at the bachelor's degree level or above. Most of the children with autism are 8–17 years old, mostly primary school students. About 40 % of the family reported a monthly income of <5,000 yuan (equivalent to 710 US dollars), below the poverty threshold (the level deemed necessary to achieve an adequate standard of living in China). Table 1 shows the demographic information of the children and their parents.

**Table 1:** Participant descriptive statistics.

**Variables**	***n***	**%**
**PARENT**		
**Gender**		
Male	113	15.9%
Female	596	84.1%
**Age**		
18–25	5	0.7%
26–30	31	4.4%
31–40	330	46.5%
41–50	292	41.2%
51–60	42	5.9%
>60	9	1.3%
**Education**		
<High school	99	14.0%
Polytechnic school or high school	122	17.2%
Mechanical degree or bachelor degree	197	27.8%
>Bachelor degree	291	41.0%
**Monthly household income (Yuan)**		
<5 K	268	37.8%
5–10 K	233	32.9%
10–20 K	114	16.1%
>20 K	94	13.3%
**Number of children**		
1	359	50.6%
2	321	45.3%
3	29	4.1%
**CHILDREN**		
**Age**		
<7	226	31.9%
8–17	410	57.8%
18–22	73	10.3%
**Education**		
Not enrolled	139	19.6%
Kindergarten	108	15.2%
Primary schools	308	43.4%
Junior high school	92	13.0%
polytechnic school or high school	54	7.6%
Specialist or undergraduate	8	1.1%

### Measures

A demographic sheet and three inventories were adopted in this study. The demographic sheet included questions to gather respondents' personal information (i.e., gender, age, employment status, educational level, family structure, monthly household income, number of children) and their children with disabilities (i.e., age, educational level). The three inventories were as follows.

#### Beach center family quality of life scale

The Beach Center Family Quality of Life Scale is a 25-item self-report measure used to examine parents' perceived Family Quality of Life [FQOL, (62)] *via* a five-point Likert-type scale (1 = very unsuitable, 2 = unsuitable, 3 = neither unsuitable nor suitable, 4 = suitable, and 5 = very suitable).

It has five subscales: (1) family interaction, reflecting the level of interaction between family members (six items); a sample item is “My family enjoys spending time together”; (2) family care and support, reflecting the level of care and attention given to raising children (six items); a sample item is “My family helps children learn to be independent”; (3) emotional happiness, reflecting the level of emotional happiness of the family (four items); a sample item for emotional happiness is “My family has friends or others to provide support”; (4) material happiness, reflecting the family's level of material wellbeing (five items); a sample item is “My family has transportation to get where they need to go”; and (5) disability-related support, reflecting the level of disability-related support received by the family (four items); a sample item is “My family member with a disability has support to accomplish goals at home”.

This scale was developed in English, translated into Chinese for this study, and then back-translated into English. In addition, the third question, “My family works together to solve problems”, and eighth question, “My family members help the children with school work and activities”, were removed from the original scale, considering the study's purpose and the local context, leaving 23 items.

**Figure 2:**
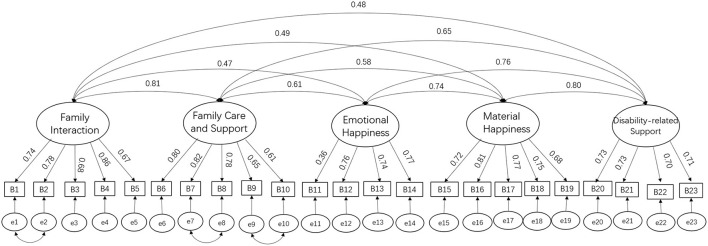
Confirmatory factor analysis of family quality of life.

After removing 52 outliers, the researchers performed a confirmatory factor analysis (CFA) on the scale for validation. The reliability and validity of the scale meet the requirements of psychometric indicators and showed good reliability and validity. As shown in Figure 2, the overall Cronbach's alpha value for the scale in this study was 0.94, with the five factors having alpha values of 0.89, 0.85, 0.75, 0.86, and 0.81, respectively, indicating a good level of internal consistency for the entire survey instrument and the five factors. The CFA results for the scale showed that the CMIN/DF was 4.41, RMSEA (Root Mean Square Error of Approximation) was 0.07, CFI (comparative fit index) was 0.92, AGFI (adjusted goodness-of-fit index) was 0.86, IFI (incremental fit index) was 0.92, and TLI (Tucker-Lewis index) was 0.91, all good indicators. The modified scale had a better fit and more desirable data than the Hoffman-designed scale.

#### Pandemic stress scale

The Psychological Stress Questionnaire (63) is a newly developed measurement that examines stress related to the COVID-19 pandemic using a five-point Likert scale. It contains nine items reflecting three factors identified by Wang J. et al. (63). The first factor is risk awareness, reflecting subjects' self-assessment of the level of risk in their environment (three items). A sample item is “What do you think is the risk of exposure to infection in your work environment?” The second factor is physical and mental response, revealing subjects' reactions to stress in the current environment (four items). A sample item is “Do you need professional psychological guidance?” The third factor relates to optimistic Hope, reflecting subjects' confidence in overcoming the pandemic and their optimism about the current pandemic attitude (two items). A sample item is “Are You confident in this anti-epidemic victory?” The “anti-epidemic victory” refers to the spread of COVID-19 across the globe, where through the widespread availability of the vaccine and the success of anti-epidemic measures, governments remove the last legal restrictions and citizens can achieve freedom of movement across regions (64).

Since latent variables need to be explained by at least three or more observed variables (65), and one factor of the Psychological Stress Scale contains only two items and is unsuitable for CFA, exploratory factor analysis (EFA) was conducted to validate the Psychological Stress Scale.

Factor analysis is appropriate when the KMO (Kaiser-Meyer-Olkin) > 0.60 and Bartlett's spherical test is statistically significant (66). Results showed that KMO = 0.83 and Bartlett sig < 0.05, indicating that the scale was suitable for EFA. Three factors were yielded *via* the component matrix. The first was *risk perception and concern* (items 2, 3, 4, 5, and 6), describing parents of children with autism's pandemic-related risk perceptions and concerns. A sample item is “you concerned about being infected during your work”. The second is *pragmatic hopefulness* (items 1 and 9), describing respondents' degree of rational attitude toward the pandemic and hope of an anti-epidemic victory. A sample item is “You are confident in this anti-epidemic victory”. The third is *physical and mental reaction* (items 7 and 8), describing respondents' physical and mental responses to the pandemic. A sample item was “you need professional psychological guidance”.

The Cronbach's alpha value of the modified scale was 0.77, indicating the whole survey instrument and the *risk perception and concern* section had good internal consistency. After maximum variance rotation, the coefficients ranged from 0.74 to 0.89 for the five risk perception and concern items, from 0.48 to 0.88 for the two *pragmatic hopefulness* items, and from −0.69 to 0.77 for the two *physical and mental reaction* items. The coefficient of item 7 was negative, while the coefficients of the other items were all positive; the item 7 scores were assigned in the reverse direction to ensure that the effect direction was consistent among all items. Based on the scale designed by J. Wang, this study adapted the structure of the EFA method model, which was an attempt to develop a pandemic stress scale, and the indicators were better than the original scale.

#### Parental involvement scale

The study drew on the Parental Involvement Scale used by Georgiou and Tourva (67), which consists of five factors: (1) involvement in school activities, reflecting how closely subjects interact with their children's school (six items). A sample item for *participation in school activities* was “Going to my child's school to talk to teachers”; (2) anxiety and overprotection, revealing specific behaviors of anxiety and overprotection in subjects' parenting (six items). A sample item was “Worrying that something bad might happen to my child”; (3) monitoring, revealing subjects' privacy and life details (six items). A sample item for *monitoring* was “Wondering who your child's friends are”; (4) homework help, reflecting subjects' involvement in their child's classroom tutoring and academic development (six items). A sample item for *homework help* is “Getting to know your child's school systematically”; and (5) interest development-extracurricular activities, reflecting subjects' involvement in their child's hobbies and interests (six items). A sample item for *interest development - extracurricular activities* is “Encouraging your child to read for pleasure”. Each item was rated on a five-point Likert-type scale (1 = very unsuitable, 2 = unsuitable, 3 = neither unsuitable nor suitable, 4 = suitable, and 5 = very suitable).

**Figure 3:**
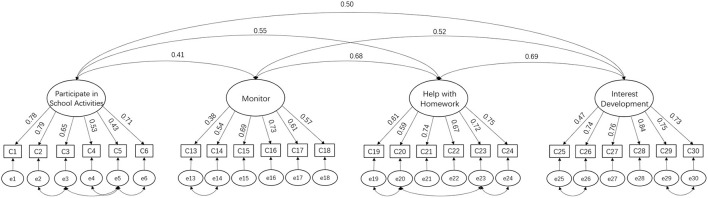
Confimatory factor analysis of parental involvement.

The *anxiety and overprotection* subscale was removed because factor loading for the anxiety and overprotection subscale (below 0.1) was too low. Furthermore, in the Chinese context, items in this subscale were not closely related to parental involvement. Furthermore, this subscale overlapped the Pandemic Stress Scale to some extent. The modified scale, shown in Figure 3, contains four factors and twenty-four items. The scale's reliability and validity meet the requirements of psychometric indicators, with good reliability and validity. The Cronbach's alpha value for the modified scale was 0.90, and the Cronbach's alpha values for the four subscales were 0.81, 0.76, 0.85, and 0.85, indicating the internal consistency levels of the whole survey instrument and the four subscales were good. CFA (using AMOS 28.0) and a chi-squared test were used to compare the fit indices of the seven models (CMIN/DF, RMSEA, GFI, AGFI, CFI, IFI, and TLI). The results showed that CMIN/DF was 3.19, RMSEA was 0.06, GFI was 0.92, CFI was 0.93, AGFI was 0.90, IFI was 0.93, and TLI was 0.92; all indices met the criteria for a good model fit. The scale was adjusted based on Georgiou and Tourva (67) to provide better data for each indicator.

### Data analysis

Data cleaning was carried out before data analysis. No variables had missing data. The outliers were processed by IBM SPSS 27.0, and 52 samples with standard scores less than or greater than 3 were removed, leaving a final sample of 709. An examination of correlations revealed that no independent variables were highly correlated (*r* > 0.80). The multi-collinearity statistics including Tolerance and VIF (variance inflation factor) were within acceptable limits.

SPSS 27.0 was used to test the three scales' reliability. SEM was used to conduct CFA on the modified Family Quality of Life Scale and Parental Involvement Scale. EFA was conducted for the Pandemic Stress Scale.

SPSS 27.0 was used to describe each FQOL subscale to answer the first research question. Then, one-sample *t*-tests were conducted to assess the mean difference between participants' perceptions of these variables and the hypothesized midpoint score (i.e., critical value = 3).

**Figure 4:**
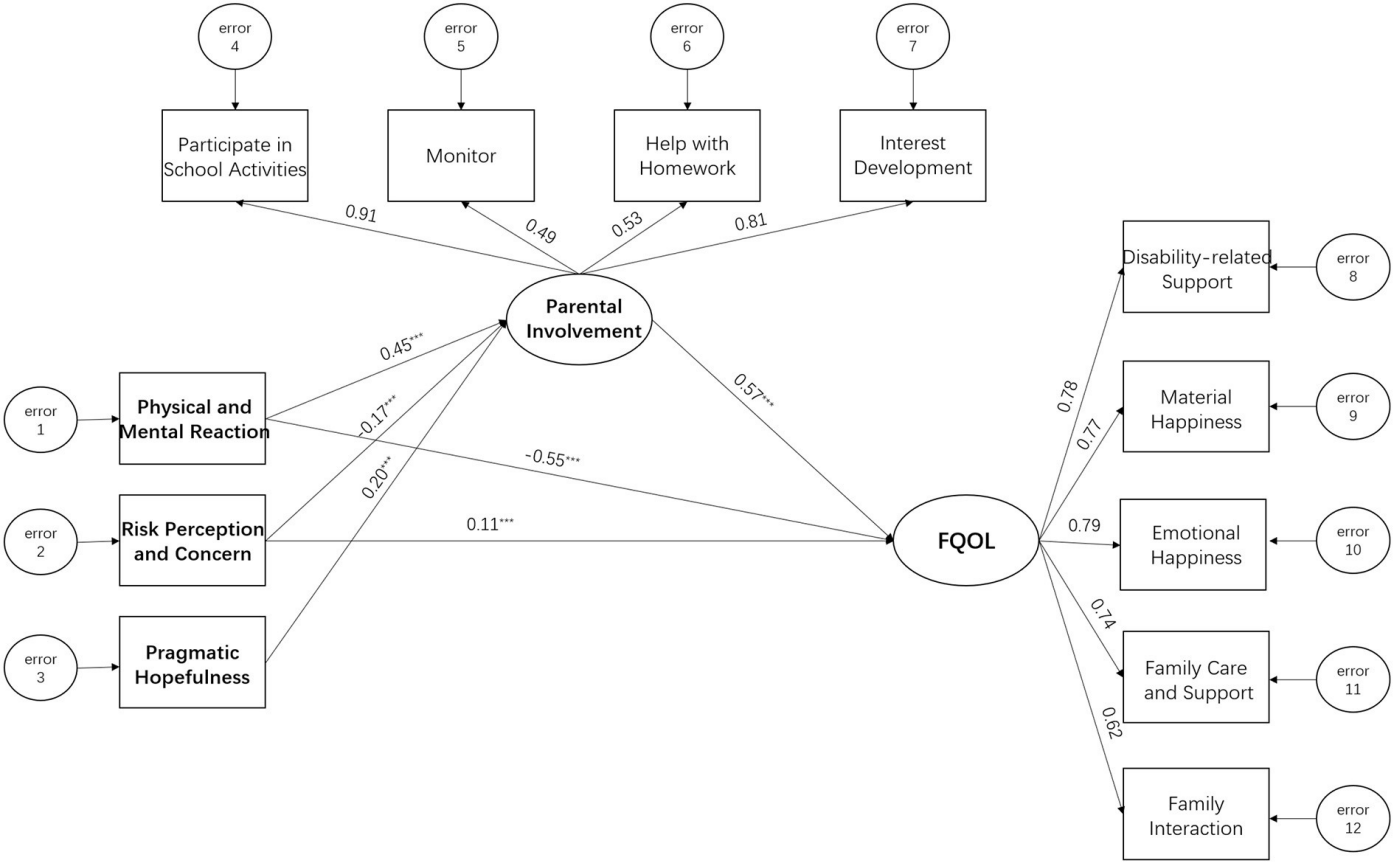
Structural equation model path diagram of the interrelations between stress, involvement, and FQOL. FQOL, family quality of life. ***p < 0.001.

Structural equation modeling (SEM) was applied to test the hypothetical model to answer the second question (see Figure 1). SEM is a series of multivariate statistical models used to estimate the effects and relationships between multiple variables representing a hypothetical, theoretical model (68). In the present research, the three variables were the sum of the items from each scale. Due to the *risk perception and concern* and *physical and mental reaction* subscales' low loading coefficients for pandemic stress and the non-significant direct effect between pandemic stress and FQOL in the hypothetical model, a new model was reconstructed, within which the three factors of pandemic stress were used as observed independent variables (see Figure 4).

## Results

### Descriptive results

The first research question addressed the current status of pandemic stress, parental involvement, and FQOL among families of children with autism in China. As shown in Table 2, the FQOL for children with autism in China is significantly higher than the expected average (score 3). *Emotional happiness* was the lowest [M = 3.12, SD = 0.81, *t*_(708)_ =3.92, *p* < 0.001], followed by *disability-related support* [M = 3.26, SD = 0.83, *t*_(708)_ = 8.33, *p* < 0.001]; the highest was *family interaction* [M = 3.91, SD = 0.77, *t*_(708)_ = 31.35, *p* < 0.001], followed by *family care and support* [M = 3.80, SD = 0.70, *t*_(708)_ = 30.55, *p* < 0.001].

**Table 2:** Descriptive statistics for family quality of life, parental involvement, and stress.

	***n***	***m***	**sd**	**df**	***T***	***p***
F1	709	3.91	0.77	708	31.35	<0.001
F2	709	3.80	0.70	708	30.55	<0.001
F3	709	3.12	0.81	708	3.92	<0.001
F4	709	3.42	0.89	708	12.61	<0.001
F5	709	3.26	0.83	708	8.33	<0.001
PS	709	3.79	0.67	708	30.97	<0.001
PM	709	3.69	0.65	708	28.37	<0.001
PH	709	3.89	0.68	708	34.88	<0.001
PI	709	4.03	0.63	708	43.67	<0.001
EW	709	2.51	0.90	708	−14.60	<0.001
EE	709	2.83	0.83	708	−5.45	<0.001
EP	709	3.91	0.66	708	37.02	<0.001

F1, family interaction; F2, family care and support; F3, emotional happiness; F4, material happiness; F5, disability related support; PS, participate in school activities; PM, monitor; PH, help with homework; PI, interest development; EE, physical and mental reaction; EW, risk perception and concern; EP, pragmatic hopefulness.

Parents' involvement with their children with autism was above our expected average (score 3). I*nterest development* was highest [M = 4.03, SD = 0.63, *t*_(708)_ = 43.67, *p* < 0.001], followed by *help with homework* (M = 3.89, SD = 0.68, *t*_(708)_ = 34.88, *p* < 0.001).

Of the three dimensions of pandemic stress, *risk perception and concern* [M = 2.51, SD = 0.90, *t*_(708)_ = −14.60, *p* < 0.001] and *physical and mental reaction* [M = 2.83, SD = 0.83, *t*_(708)_ = −5.45, *p* < 0.001] were lower than the expected mean, while *pragmatic hopefulness* was higher [M = 3.91, SD = 0.66, *t*_(708)_ = 37.02, *p* < 0.001].

### SEM results

The second research question concerned the relationship between pandemic stress, parental involvement, and FQOL. The final SEM (see Figure 4) was determined and the model fit index indicated it was feasible, where X^2^/df = 13.40, RMSEA = 0.13, GFI = 0.86, AGFI = 0.80, CFI = 0.78, IFI = 0.78, and TLI = 0.72.

The modification indices function was used to see if Amos could propose further improvements to the model. After the modification, the SEM has a better model fit index, X^2^/df = 5.323, RMSEA = 0.08, GFI = 0.95, AGFI = 0.91, CFI = 0.94, IFI = 0.94, TLI = 0.90. In addition, by conducting exploratory model exploration with Amos, the output suggested deleting the direct covariate path between *pragmatic hopefulness* and FQOL; the current model was the preferred SEM for revealing these data and describing the correlation effects between pandemic stress, parental involvement, and FQOL.

The range of standardized loadings for each latent variable and the observed scales and standardized path coefficient is shown in Figure 4. FQOL ranged from 0.62 to 0.79 and parental involvement ranged from 0.49 to 0.91, with all loading indices >0.40 and statistically significant (*p* < 0.001), indicating that the dimensions of the factors adequately measured and explained the latent variables.

The direct effect of *pragmatic hopefulness* on FQOL was not significant and had a positive effect on FQOL through the mediation of parental involvement as shown in Table 3, a full mediation effect with an effect size of 0.20^*^0.57 = 0.114. *Risk perception and concern* had a direct positive effect on FQOL with a size of 0.11 and a negative effect on FQOL through the mediation of parental involvement with a size of −0.17^*^0.57 = −0.097. The total effect of *risk perception and concern* on FQOL is positive, size 0.11–0.17^*^0.57 = 0.013; *physical and mental reaction* had a direct negative effect on FQOL, size −0.55, a positive effect on FQOL mediated through parental involvement, size 0.45^*^0.57 = 0.257, a negative total effect on FQOL with a size of −0.55 + 0.45^*^0.57 = −0.293.

**Table 3:** Results of structural equation model analysis.

**Model**	**EE**	**EW**	**EP**	**Involvement**
**Direct effects**				
FQOL	−0.55^***^	0.11^***^		0.57^***^
**Indirect effects**				
Involvement	0.45^***^	−0.17^***^	0.20^***^	
FQOL	0.257^***^	−0.097^***^	0.114^***^	
**Total**				
FQOL	−0.293^***^	0.013^***^	0.114^***^	

EE, physical and mental reaction; EW, risk perception and concern; EP, pragmatic hopefulness; FQOL, family quality of life.

*** *p* < 0.001.

To summarize, the model results largely supported the two hypotheses. Consistent with the first hypothesis, there was a significant direct correlation between pandemic stress and FQOL. The direct predictive effect of pandemic stress on FQOL was supported by two dimensions, *physical and mental reaction* and *risk perception and concern*, where *physical and mental reaction* was negatively related to FQOL and *risk perception and concern* was positively related to FQOL. Consistent with the second hypothesis, there was a significant indirect correlation between pandemic stress and FQOL, mediated by parental involvement. The three dimensions of *physical and mental reaction, risk perception and concern*, and *pragmatic hopefulness* supported the indirect predictive role of pandemic stress on FQOL, where *physical and mental reaction* was positively related to FQOL, *risk perception and concern* was negatively related to FQOL, and *pragmatic hopefulness* was positively related to FQOL.

## Discussion

This study aims to describe the current status of pandemic stress, parental involvement, and family quality of life for children with ASD, and explore the relationships among the three variables. Results responded well to the research questions, and the research hypotheses were largely supported.

### The current status of pandemic stress, parental involvement, and FQOL

Families of children with ASD had relatively higher satisfaction with *family interaction* and relatively lower satisfaction with their e*motional wellbeing*, which is consistent with previous research (69). On the one hand, this may be due to the fact that many people in China consider ASD as a stigma (70), and social labeling and self-labeling reduce their self-identity and emotional level needs. On the other hand, studies have showed that family quality of life for children with ASD decreases under the psychological stress [e.g., (5)]. Family quality of life for children with ASD was significantly influenced by the pandemic. For parents of children with ASD, their interaction with the surrounding environment was reduced, and consequently social inclusion was hindered as well as emotional needs were unmet.

During the COVID-19, parents of children with special needs spent more time and energy caring for their children because of the limitations of pandemic prevention regulations such as isolation (34–36). In China, parents of children with ASD are often involved in all aspects of their children's learning and life due to their children's medical condition. Results showed that parents were sufficiently involved in all four areas, with the highest involvement being in “interest development-extracurricular activities”, which is consistent with previous studies (67, 71). Meanwhile, the lowest level of involvement was “monitoring”, which is consistent with several studies on parental involvement in cerebral palsy, surgical hospitalization, and mobile children (72–74).

### The relationships among pandemic stress, parental involvement and FQOL

Previous studies have confirmed the positive predictive effect of parental involvement behaviors on FQOL (12, 50, 58), and promoting parental involvement in the learning, living, and rehabilitation interventions of children with ASD is beneficial not only for the development of children with ASD, but also for their family life situation.

For *physical and mental reaction*, the SEM results show a direct negative effect of *physical and mental reaction* on family quality of life and a positive effect on family quality of life mediated by parental involvement, with a negative overall effect of *physical and mental reaction* on family quality of life. Higher *physical and mental reaction* means parents of children with ASD experience more physical and mental suffering in pandemic, such as insomnia, and have more need for counseling caused by high level anxiety. Family quality of life is a multidimensional concept that involves people's emotional wellbeing, and the *physical and mental reaction* to the pandemic reduce people's emotional wellbeing, which in turn reduces the quality of family life.

*Risk perception and concern* had a direct positive effect on family quality of life and a negative effect on family quality of life mediated through parental involvement, with a small positive total effect on family quality of life. This is also inconsistent with previous results regarding the negative effect of stress on family quality of life (7, 8, 24, 25). Considering the pandemic, the more severe the perception of the pandemic, the stronger the desire to reduce the impact of the pandemic on children with ASD in various ways, and the increased family care, material wellbeing and disability-related support, which would improve their quality of life; at the same time, *risk perception and concern* of the pandemic may discourage parents from participating in their children's lives and thus reduce their participation behavior, but overall, *risk perception and concern* during the pandemic enhance family quality of life to some extent.

Although *pragmatic hopefulness* has no direct effect on FQOL, it has positive indirect effect on FQOL through parental involvement. The higher level of *pragmatic hopefulness* results in higher parental involvement, and the higher level of parental involvement results in higher level of FQOL. This means the increase of *pragmatic hopefulness* can improve FQOL for children with ASD. The continued spread of pandemic and the associated home isolation requires pragmatic hope so that families of children with autism could mobilize their own resources for a more active life. Providing social support to families of children with autism to help them develop an objective and positive intellectual orientation to the outbreak is critical (56), and the public health sector should provide families of children with autism with timely and correct knowledge and guidance about the impact of the outbreak on their personal health as well as on their child's growth and development (4, 21).

It is worth exploring that as a dimension of pandemic stress, *physical and mental reaction* elevate parental involvement, which is contrary to findings of previous research (55, 56). This may be related to the context of the pandemic. Parents reacted strongly both physically and mentally during the pandemic and were more concerned about their children, minimizing the impact on their children by over-caring for them. At the same time, the policy of home isolation in pandemic situations requires greater parental control of children in the home, and school rules for online teaching make parents more participating in their children' schooling actively or passively.

## Limitations

The current study has five limitations. First, pandemic stress was initially constructed as a second-order latent variable, but it is not so fit in original SEM model, and to reconstruct the model, three factors of pandemic stress was used as observed variables, respectively. The relationships abovementioned need verification in the future study. Second, the model did not incorporate covariates, such as gender, household income status, and other demographic contexts. Most participants were mothers, but the influence of fathers was equally important and needed to be considered and discussed. There may be subgroup differences in family quality of life for families with different income levels. In addition, 73 participants aged from 18 to 22 (only 10%) were included in the present research, which may have influenced the present study's results.Third, the study attempted to obtain a diverse sample in mainland China, but the representativeness of the sample needs further verification as it currently lacks the support of a national census. Fourth, the data collected in this questionnaire are cross-sectional in nature. A longitudinal design may be conducted in the future, which can better argue the cause-effect of pandemic pressure on parental involvement and FQOL. Fifth, this study adopted online survey, which might have inadvertently excluded some parents such as low-income, resource-constrained single-parent groups, and those without smartphones/laptops/tablets.

## Significance and implication

The present study makes three important contributions. First, this is the pioneer study to investigate the impact of COVID-19 pandemic on parenting children with ASD in mainland China which has a large number of children with ASD and is highly impacted during COVID-19 pandemic in terms of life and schooling. Second, the study explored the pandemic stress among parents of children with ASD during COVID-19, which enriched the research field of pandemic stress. Furthermore, the study verified the mediating role of parental involvement and enriched the research related to the relationship between parental involvement and family quality of life (FQOL). Finally, this study enriched the databank regarding the psychometric properties of the three scales and tested the Pandemic Stress Scale, the Parental Involvement Scale, and the FQOL Scale. Notably, this is the pioneer study using EFA to explore pandemic stress among children with ASD in a Chinese context.

Reference can be made to the findings of this study to pay attention to parents' *risk perception and concern, physical and mental reaction*, and *pragmatic hopefulness* during COVID-19 to better promote parental involvement in the life, learning, and rehabilitation of children with ASD and to enhance family quality of life for children with ASD. Three detailed practical implications may be proposed. First, a systematic psychological intervention services may be provided for family of children with ASD to reduce their physical and mental response to the pandemic, and thus to enhance the overall FQOL. For instance, rational emotive behavior therapy could help parents reduce their psychological stress. Specifically, the ABC model encourages parents to look at the “activating event” (e.g., their goals and difficulties) and “emotional disturbance” (their own largely negative “beliefs” or interpretations of these events) they have experienced. Afterwards, attention is directed to the “beliefs” and inferences that powerfully influence emotional disturbance. It is possible to teach this model effectively and quickly, and most parents can grasp it, apart from those who are seriously ill or confused. Parents are encouraged to learn relaxation procedures, yoga or meditation, as well as how to dispute problem-causing irrational beliefs.

Second, parent-to-parent groups have played important roles in promoting parental adaptation (75), so family support groups to reduce the pandemic stress are also one of the most important ways to improve the family quality of life. In addition, ASD caregivers need more support during the pandemic. Caregivers of children with ASD can learn behavioral strategies and interventions through telehealth training programs to help reduce their stress and improve their wellbeing during the COVID-19 pandemic (76).

Third, interventions with families of children with ASD have had a positive effect in improving family quality of life of caregivers' families, but these strategies are still in their infancy and need to be further explored, especially given the complexity of the pandemic (76, 77). At the policy level, during the COVID-19 and similar pandemic, taking effective actions to maintain and enhance the pragmatic confidence of people who are involved in is very important to encourage parental involvement, and thus to maintain and improve their family quality of life. For instance, university/school administrators could offer resources and inform parents of various involvement strategies, or compare data on university/school-level parental involvement across districts; if university/school-level parental involvement is low, counselors could work with administrators and parent-teacher associations to create more welcoming and inviting environments and provide more opportunities for parents to engage in university/school activities—e.g., by arranging flexible times for working parents to attend parent-teacher meetings and other events inside and outside the classroom or providing child care or refreshments at evening events (78).

## Data availability statement

The raw data supporting the conclusions of this article will be made available by the authors, without undue reservation.

## Ethics statement

The studies involving human participants were reviewed and approved by the Ethics Committee of Shandong University. The patients/participants provided their written informed consent to participate in this study.

## Author contributions

ShC supervised the whole manuscript. SaC was responsible for conceptionalization and finalize the manuscript. SL analyzed the data. YL drafted the manuscript. All authors contributed to the article and approved the submitted version.

## Funding

This manuscript was supported by the Qilu Young Scholars Discipline Construction Fund of Shandong University (No. 11090082163149).

## Conflict of interest

The authors declare that the research was conducted in the absence of any commercial or financial relationships that could be construed as a potential conflict of interest.

## Publisher's note

All claims expressed in this article are solely those of the authors and do not necessarily represent those of their affiliated organizations, or those of the publisher, the editors and the reviewers. Any product that may be evaluated in this article, or claim that may be made by its manufacturer, is not guaranteed or endorsed by the publisher.
